# A review of sex-related differences in colorectal cancer incidence, screening uptake, routes to diagnosis, cancer stage and survival in the UK

**DOI:** 10.1186/s12885-018-4786-7

**Published:** 2018-09-20

**Authors:** Alan White, Lucy Ironmonger, Robert J. C. Steele, Nick Ormiston-Smith, Carina Crawford, Amanda Seims

**Affiliations:** 1 Institute of Health & Wellbeing, Leeds Beckett University, Civic Quarter, Leeds, LS1 3HE UK; 2 Cancer Research UK, Angel Building, 407 St John Street, London, EC1V 4A UK; 3 Division of Cancer Research, Centre for Research into Cancer Prevention and Screening (CRiPS), University of Dundee, Dundee, DD1 9SY UK; 4 Department of Health, 15 Butterfield Street, Herston, Brisbane, 4006 QLD Australia

**Keywords:** Colorectal Cancer, Premature death, screening, Sex/gender difference, Routes to diagnosis, Staging, survival

## Abstract

**Background:**

Colorectal cancer (CRC) is an illness strongly influenced by sex and gender, with mortality rates in males significantly higher than females. There is still a dearth of understanding on where sex differences exist along the pathway from presentation to survival. The aim of this review is to identify where actions are needed to improve outcomes for both sexes, and to narrow the gap for CRC.

**Methods:**

A cross-sectional review of national data was undertaken to identify sex differences in incidence, screening uptake, route to diagnosis, cancer stage at diagnosis and survival, and their influence in the sex differences in mortality.

**Results:**

Overall incidence is higher in men, with an earlier age distribution, however, important sex differences exist in anatomical site. There were relatively small differences in screening uptake, route to diagnosis, cancer staging at diagnosis and survival. Screening uptake is higher in women under 69 years. Women are more likely to present as emergency cases, with more men diagnosed through screening and two-week-wait. No sex differences are seen in diagnosis for more advanced disease. Overall, age-standardised 5-year survival is similar between the sexes.

**Conclusions:**

As there are minimal sex differences in the data from routes to diagnosis to survival, the higher mortality of colorectal cancer in men appears to be a result of exogenous and/or endogenous factors pre-diagnosis that lead to higher incidence rates. There are however, sex and gender differences that suggest more targeted interventions may facilitate prevention and earlier diagnosis in both men and women.

## Background

Colorectal cancer (CRC) is the UK’s third most common cancer in men (after prostate and lung cancer) and women (after breast and lung cancer) [1] costing the UK approximately £1.6bn [2]. CRC is a disease that has both biological sex differences and socio-cultural gender components [3–10]. Greater awareness of how sex and gender impact on CRC may therefore lead to new insights into how improvements in prevention, early diagnosis, treatment and survival can be made.

More males develop CRC, with age-standardised rates (ASRs) of 86.1 per 100,000 males compared to 56.9 per 100,000 female in the UK in 2014 (which equates to 22,844 male and 18,421 female new cases annually) [11]. CRC mortality rates are also higher in men (ASRs of 33.9 per 100,000 males c.f. 21.8 per 100,000 females) [11]. Mortality rates are significantly higher for males than for females in all age groups from 45 to 49 and over, and the gap is widest at the ages of 70–74, when the male:female age-specific mortality rate ratio is around 1.7:1 [11]. There is also a global trend for men to have both higher incidence (746,298 vs 614,304 [20.6 vs14.3 ASR]) and mortality (373,639 vs 320,294 [10 vs 6.9 ASR]) for CRC [12].

Previous research has reported that sex differences in CRC exist with regard to its type [13], location [5, 14, 15] and survival [16] and in the health behaviour of men and women with regard to lifestyle-related risk factors [17–20], awareness of risk [21], and screening behaviour [22, 23]. This review however, critically explores sex differences for data across the CRC cancer pathway (screening uptake, the route to diagnosis, cancer staging at diagnosis and survival), and how they might play a part in the sex differences in mortality. These differences are explored alongside a review of the literature on biological awareness and behaviour differences to give direction towards strategies to increase early detection and reduce cancer burden and mortality.

## Method

The data used for the review comprised a cross-sectional study of CRC, compiling national data available for the UK (or for England where UK data for all countries combined were not available). Data were sourced from a range of publicly available datasets (or via personal communication where possible where data were not published), and peer reviewed literature. The following metrics and sources were used:

- CRC incidence rates, by age (2012–2014) and by anatomical site (2010–2012) in the UK [11]
- Screening uptake and positivity rates of the guaiac-based faecal occult blood test (gFOBT), for 60–74 years olds combined and by five-year age band, in England 2014–2015 [24]
- Routes to diagnosis data which show the proportion of CRC patients who were diagnosed through the different pathways up to the point of diagnosis, England 2006–2013 [25]
- Stage at diagnosis data, England 2012 [26]
- Stage at diagnosis by route to diagnosis 2012–2013 [27]
- 1 and 5-year age-standardised net survival for patients diagnosed in England 2011–2015 [28]
- Age-standardised net survival by stage at diagnosis: one-year survival for patients diagnosed in England 2012–2014 [29], 5-year survival for patients diagnosed in the former Anglia Cancer Network area 2006–2010 [30]

Where available, 95% confidence intervals (CIs) were used as per the CIs provided by data sources, and examined for overlap to identify statistically significant differences between sexes. Where CIs were not provided (screening uptake/positivity rates and proportions by stage at diagnosis), the two-sample test of proportions was used to identify statistically significant differences between sexes.

## Results

### Incidence of Colorectal cancer

**Fig. 1:**
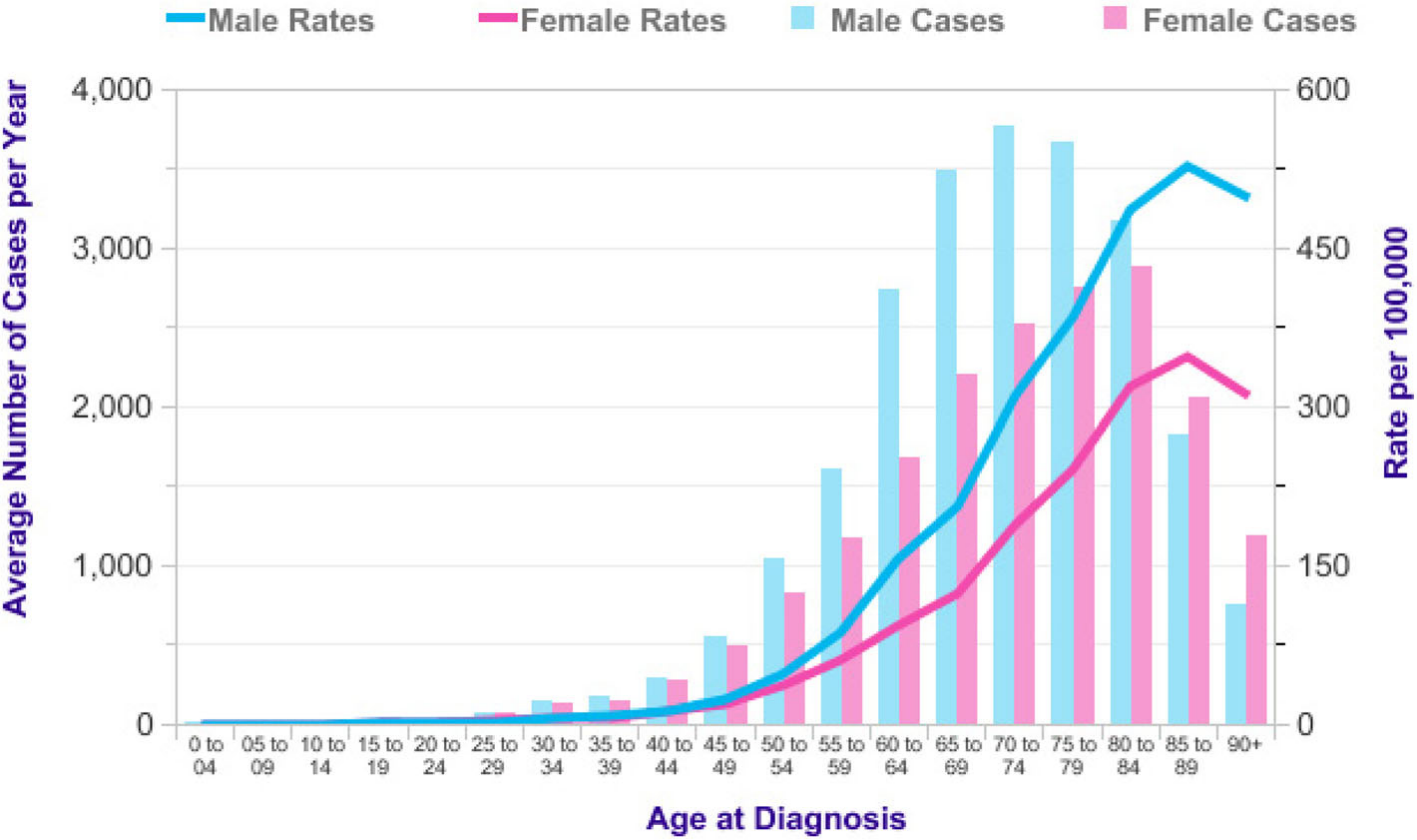
Bowel Cancer (C18-C20): 2012–2014. Average Number of New Cases Per Year and Age-Specific Incidence Rates per 100,000 Population, UK [11] (with permission to publish from CRUK)

In the UK, 2012–2014 incidence rates in adults aged 45 and over were significantly higher for males than females and this gap was widest at ages 70–74 where the male:female incidence ratio of age-specific rates was 1.7:1 [11] (Fig. 1).

A breakdown of CRC incidence by anatomical site (Table 1) shows that the proportions of CRC cases in the rectum and sigmoid colon are higher in males (31.5% and 23.1%, respectively) than females (23.1% and 20.4%, respectively). The proportion of cases in the caecum and ascending colon are higher in females (17.2% and 9.8%, respectively) than males (12.2% and 7.3%, respectively) [11], which are harder to detect and diagnose.

**Table 1:** Percentage distribution of cases diagnosed by anatomical site, by sex, UK, annual average 2010–2012 [11]

Cancer site (ICD-10 code)	Male		Female	
	Average Cases	%	Average Cases	%
Caecum (C18.0)	2829	12.2%	3145	17.2%
Appendix (C18.1)	254	1.1%	356	1.9%
Ascending Colon (C18.2)	1691	7.3%	1790	9.8%
Hepatic Flexure (C18.3)	673	2.9%	616	3.4%
Transverse Colon (C18.4)	1098	4.7%	1148	6.3%
Splenic Flexure (C18.5)	539	2.3%	393	2.1%
Descending Colon (C18.6)	707	3.0%	558	3.0%
Sigmoid Colon (C18.7)	5380	23.1%	3740	20.4%
Colon, Overlapping and Unspecified (C18.8-C18.9)	1153	5.0%	1243	6.8%
Rectosigmoid Junction (C19)	1631	7.0%	1089	5.9%
Rectum (C20)	7327	31.5%	4240	23.1%
Total	23,282	100.0%	18,317	100.00%

### Screening

Overall screening uptake is higher in women than men; for the financial year 2014–15, uptake of gFOBT screening in England (Table 2) was higher amongst women aged 60–74 (60.9%) than men (55.5%) (*p* < 0.001). By 5-year age group: uptake is higher for women aged 60–64, 65–69 and 70–74 compared with men in the same age bands (all *p* < 0.001), but this gap appears to narrow with age. Positivity rates (proportion of adequately completed gFOBT tests coming back as ‘positive’/‘abnormal’) are higher in men than women (p < 0.001), with the total positivity rate for men 2.2% and for women 1.5% in the 60–74 age range [24].

**Table 2:** Screening uptake and positivity rates, England, financial year 2014/15 [24]

	Age	Number invited	Uptake	Positivity rate
Female	60–64	893,199	58.1%	1.4%
	65–69	687,690	64.3%	1.4%
	70–74	524,607	61.1%	1.7%
	Total 60–74	2,105,496	60.9%	1.5%
Male	60–64	885,340	49.8%	2.1%
	65–69	649,180	60.1%	2.1%
	70–74	477,750	59.8%	2.4%
	Total 60–74	2,012,270	55.5%	2.2%

Uptake: proportion of invitees who were adequately screened (reaching a definitive gFOBt outcome of either ‘Normal’ or ‘Abnormal’/‘Positive’) within 6 months of invitation

Positivity rate: proportion of those adequately screened who had an abnormal/positive screening test result

### Route to diagnosis

There are interesting sex differences demonstrated in the pathway to the point of diagnosis of men and women. For cancers diagnosed in England in 2006–2013, a higher proportion of CRCs in males were diagnosed via the bowel screening program (8.1% of male CRCs c.f. 5.1% of female CRCs) and the “Two Week Wait” (TWW) pathway for urgent GP referrals for suspected cancer (29.0% c.f. 26.6%) (Table 3) [24]. Both these routes are associated with a higher three-year relative survival than average across all routes for CRC.

**Table 3:** Routes to diagnosis, by sex and age: colorectal cancer, England 2006–2013 [25]

Age group	Sex	Route								
		Screening	TWW	GP referral	Other outpatient	Inpatient elective	Emergency presentation	DCO	Unknown	Total cases
Under 50	Female	0.1%	17.2%	29.5%	9.8%	6.2%	31.3%	0.1%	5.9%	6847
	Male	0	19.6%	29.1%	9.7%	7.4%	27.3%	0.1%	6.8%	7356
50–59	Female	0.1%	30.3%	29.0%	8.6%	6.0%	20.8%	0.2%	5.1%	11,728
	Male	0.0%	31.7%	28.0%	8.4%	6.5%	19.3%	0.1%	5.9%	16,027
60–69	Female	17.7%	26.6%	21.9%	7.6%	4.2%	18.4%	0.1%	3.4%	25,288
	Male	21.9%	27.3%	20.6%	7.1%	4.3%	15.5%	0.2%	3.2%	40,734
70–79	Female	4.1%	30.9%	25.9%	8.8%	4.0%	23.5%	0.2%	2.7%	34,430
	Male	5.9%	32.1%	25.8%	9.0%	4.3%	20.6%	0.2%	2.2%	48,055
80–84	Female	0.2%	27.6%	25.3%	8.6%	3.5%	31.8%	0.5%	2.5%	18,526
	Male	0.5%	30.5%	26.5%	9.1%	3.7%	27.3%	0.3%	2.0%	19,508
85+	Female	0.1%	19.7%	20.9%	6.7%	2.7%	44.8%	1.9%	3.2%	20,516
	Male	0.1%	23.6%	23.8%	7.7%	3.0%	38.5%	0.9%	2.4%	14,865
All ages	Female	5.1%	26.6%	24.6%	8.2%	4.1%	27.6%	0.5%	3.3%	117,335
	Male	8.1%	29.0%	24.6%	8.3%	4.5%	22.1%	0.3%	3.1%	146,545

There was also a sex difference for emergency presentations (EPs), the route associated with the worst survival, with more female CRCs being diagnosed via this route than males (27.6% female CRCs c.f. 22.1% male CRCs). This difference was greatest for patients aged 85+, with 44.8% of female cases aged 85+ diagnosed through EPs compared with 38.5% of males.

### Sex differences in stage of cancer at diagnosis

There are small differences in the stage distribution of CRCs at diagnosis between males and females (Table 4) [26]. When excluding cases with an unknown stage from the denominator, data for England in 2012 show a higher proportion of CRCs in males diagnosed at stage I than females (18.2% in males c.f. 16.3% in females; *p* < 0.001). Conversely, more females than males were diagnosed at stage II (28.7% in females c.f. 27.1% in males; p < 0.001). When stage I and II are combined, there was no longer a difference between males and females. There were no differences between the proportions of males and females diagnosed at stages III and IV, both separately and combined (stage III *p* = 0.25, stage IV *p* = 0.08, combined *p* = 0.65). Staging completeness was higher for males than females (90.0% c.f. 87.7%; p < 0.001).

**Table 4:** TNM stage of colorectal cancer at diagnosis by sex, adults aged 15–99, England, 2012 [26]

Stage	Number of cases		Proportion of cases		Proportion of cases excluding unknows	
	Males	Females	Males	Females	Males	Females
I	3144	2111	16.4%	14.3%	18.2%	16.3%
II	4680	3722	24.4%	25.2%	27.1%	28.7%
III	5336	3922	27.8%	26.5%	30.9%	30.2%
IV	4136	3215	21.5%	21.7%	23.9%	24.8%
Unknown	1919	1826	10.0%	12.3%	–	–
Total	19,215	14,796	100.0%	100.0%	100.0%	100.0%

**Fig. 2:**
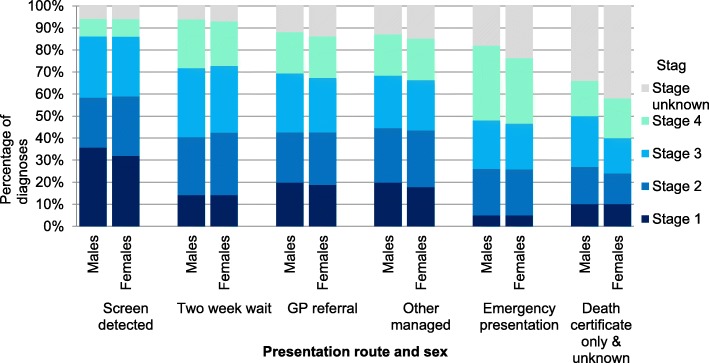
Stage at diagnosis by route to diagnosis, adults aged 15–99, England, 2012–2013 [27] (‘Other managed’ includes ‘other outpatient’ and ‘inpatient elective) (with permission to publish from CRUK)

To explore this further, the routes to diagnosis data have been combined with staging data to see how stage distributions vary by route (Fig. 2) [27]. For patients diagnosed in England in 2012–2013, 32% of all patients diagnosed with CRC via an EP were diagnosed at stage IV, with this proportion varying between the sexes (Fig. 2). More male EPs were diagnosed at stage IV than female (34% males (33–35%) *c.f.* 30% (29–31%) females) such that even though more females present via an EP, males are more likely to be diagnosed at a late stage via this route. This might explain the finding of no overall difference in stage at diagnosis between the sexes despite higher EP rates in women. However, a higher proportion of female cases had an unknown stage, so difference in stage by route should be interpreted with caution.

Out of those diagnosed via screening, more males were diagnosed at stage I [36% males (34–37%), 32% (30–34%) females], while females were more likely to be diagnosed at stage II [27% (26–29%), females, 23% (22–25%) males]. There were no other differences in stage distribution between sexes across the other routes analysed.

### Survival

Overall, age-standardised one-year net CRC survival is slightly higher for males, whilst five-year net survival is similar for the sexes (Table 5). By age group, one-year net survival for CRC is similar for males and females across all ages, except for the 65–74 and 75–99 year age groups where males have a slight advantage over females (82.7% cf. 81.3% and 67.9% cf. 62.3%). Five-year net survival data are similar between males and females for all ages, except 75–99 (49.0% cf. 46.4%).

**Table 5:** One-year and five-year net survival, with 95% confidence intervals (CI), for colorectal cancer, for adults aged 15–99 diagnosed during 2011–2015, England, by age and sex [28]

Sex	Age group	Number of patients	One-year survival			Five-year survival		
			%	95% CI		%	95% CI	
Men	Age-standardised	91,876	79.4	79.2	79.7	60.8	60.3	61.3
	Un-standardised	91,876	77.7	77.4	78	59.4	58.9	60.0
	15–44	2887	87.6	86.4	88.8	70.7	68.6	72.8
	45–54	6214	86	85.1	86.8	63.4	61.9	64.9
	55–64	17,462	85.8	85.2	86.3	67.2	66.3	68.1
	65–74	28,572	82.7	82.2	83.1	66.1	65.3	66.9
	75–99	36,741	67.9	67.4	68.5	49	48	50.1
Women	Age-standardised	73,104	77.3	77	77.6	60.1	59.6	60.6
	Un-standardised	73,104	73.7	73.4	74	57	56.5	57.6
	15–44	2966	88.7	87.5	89.8	71.3	69.3	73.3
	45–54	5250	86.7	85.7	87.6	66.5	64.9	68.1
	55–64	11,617	86	85.3	86.6	67.9	66.8	69
	65–74	18,637	81.3	80.7	81.9	65.1	64.2	66.1
	75–99	34,634	62.3	61.7	62.8	46.4	45.5	47.3

One-year age-standardised net survival at stage I and II estimates for patients diagnosed in England in 2014 are similar for males and females (Table 6). Females diagnosed at stage III and IV and unknown stage have worse survival than males.

**Table 6:** One-year age-standardised net survival, with 95% confidence intervals (CI), for colorectal cancer by TNM stage at diagnosis, for adults aged 15–99 diagnosed in England during 2014, by sex [29]

Colorectal		All stages		Stage I		Stage II		Stage III		Stage IV		Unknown	
By sex	Male	77%		98%		93%		89%		44%		57%	
	Confidence interval	77%	78%	96%	98%	92%	93%	88%	90%	42%	45%	56%	59%
	Number in cohort	18,489		2990		4297		4970		4244		2073	
	Female	73%		98%		91%		85%		35%		50%	
	Confidence interval	72%	73%	97%	99%	90%	92%	84%	86%	34%	37%	49%	52%
	Number in cohort	14,905		2213		3576		3830		3353		2013	

Five-year age-standardised net survival was similar between males and females for stages I, III and IV (Table 7), but survival at stage II was better for females (87% cf. 82% males) [30].

**Table 7:** Five-year net survival by stage, with 95% confidence intervals (CIs), for colorectal cancer, for adults aged 15–99 diagnosed in the former Anglia Cancer Network during 2006–2010, by sex [30]

	5 Year Survival					
	Male (%)	95% CI		Female (%)	95% CI	
Stage I	94	91	97	97	95	100
Stage II	82	80	84	87	85	89
Stage III	60	57	63	61	59	64
Stage IV	7	6	9	8	6	10
All stages	61	60	63	63	62	65

## Discussion

The UK data reflect previous studies [15, 31, 32] in showing that the overall incidence of bowel cancer is higher in males than in females. This increased vulnerability of men to developing CRC may be due to a number of biological and gender-related (behavioural) factors [31, 33–35]. Men are more likely to have a diet high in red and processed meat [36], be heavier consumers of alcohol [37], and more likely to smoke [20]. Men also have a greater propensity to deposit visceral fat [38] which is associated with increased risk of CRC [39–41].

It is important to note, however, that the female sex is more associated with hypermethylation, microsatellite instability, BRAF V600E mutation, and CpG island methylator phenotype (CIMP)-high, [7, 13], which are more likely to result in the sessile serrated polyps (SSP). These occur in the proximal colon and are more likely to be missed during colonoscopies and lead to more aggressive forms of cancer [14]. Females were also found to have higher frequency of KRAS mutations in codon 12 than males, which again are associated with more advanced adenomas [42].

Socio-economic deprivation (as based on income domain scores) appears to disproportionally affect the incidence rates for men - in England, incidence rates are 13% higher for males living in the most deprived areas compared with the least deprived, while for females the rates are similar for those living in the least and most deprived areas [43]. This may be due to men’s increased likelihood of a lifestyle associated with the risk factors mentioned above in areas of socio-economic deprivation [44, 45].

### Screening

A factor relating to men’s relatively lower participation, especially with regard to their higher overall risk of CRC, in screening has been attributed to poorer knowledge about cancer and screening as compared to women, with the 2014 Cancer Awareness Measure (CAM) survey reporting that men were less likely to be aware of the bowel screening programme than women (*p* < 0.05) [46]. This lack of awareness has been noted elsewhere, both with regard to men’s awareness of screening generally [47, 48] and CRC specifically [10, 49].

Encouraging men to discuss bowel screening with their GP or partner on receipt of an invitation may improve uptake [50] - the role of primary care in nudging both men and women to take up cancer screening opportunities has been noted elsewhere [49, 51–53]. Sending enhanced information associated with the national screening programme has been shown to increase uptake of gFOBT in both sexes [54] and an Australian study showed sending men a notification letter prior to faecal immunochemical test (FIT) screening resulted in a 12% increase in uptake compared to those who were not contacted in advance [55].

The UK National Screening Committee has recommended the change from using gFOBT to the less onerous FIT as the primary test for bowel cancer screening in the UK - pilot studies show this improves uptake [22, 56] and reduces the male-female gap in uptake compared to gFOBT [57]. It is imperative that there is a timely roll out of FIT across the UK countries to benefit both men and women as soon as possible, and reduce the sex difference.

The recently introduced bowel scope screening (BSS) in England at the age of 55 overcomes some of the issues associated with faecal sampling, but retains some gender specific issues with regard to its acceptability and uptake. It has been shown that women are less likely than men to take up the opportunity [58, 59], with anticipated greater discomfort, anxiety and embarrassment from the test, and the gender of the practitioner undertaking the test seeming to be particular barriers [60]. However, uptake in women has been shown to be significantly greater compared to men when invited to self-refer (20.7% vs 8.8%; X^2^-test of independence - *P* = 0.05) which may have been facilitated by their opportunity to request a same-sex practitioner (requested by 100% of female attendees vs 67% of males *P* < 0.05) [61], highlighting the importance of screening programmes to acknowledge gender specific barriers.

### Sex differences in the effectiveness of screening

Although women are more likely to accept an invitation to screening using gFOBT, they seem to benefit less as the number needed to screen to detect a CRC is higher in women than in men at all ages [62]. In part, this can be explained by the discrepancy in incidence between the sexes at the screening ages, but it is also now well established there are more interval cancers in women, indicating that gFOBT is less sensitive in women than in men [63]. The reason for this latter observation is not clear, but initial work on quantitative FIT for haemoglobin has demonstrated very clearly that women have significantly less haemoglobin in faeces than do men [64, 65]. Thus, if there was an aim to equalise the screening positivity rate in men and women, it would be necessary to set different thresholds for faecal haemoglobin (FHb) concentration. However, the positive predictive value (PPV) for women is less than for men, so that were a lower cut-off for triggering an invitation to colonoscopy used in women, a few more cancers might be detected at screening, but at the cost of many more negative colonoscopies. The balance between benefit and harm would therefore be altered by taking such an approach, and this has not been adequately modelled. In the future however, a risk score based on FIT-derived FHb, but incorporating sex and age, might address this issue.

It is not known why women have lower levels of FHb, and why FHb has a lower PPV for colorectal neoplasia, but it has been demonstrated that FHb concentration is strongly correlated to both all cause and non-colorectal cancer mortality [66], and may be acting as a non-specific marker for health status. This is in keeping with the observations mentioned above, that women tend to have a healthier lifestyle and are less prone to the deposition of visceral fat than men.

### Route to diagnosis

Females are typically a decade older when they develop the condition, with a higher risk of co-morbidity [7], which masks symptoms and more negatively affects their survival and may explain why they have higher rates of EP. However, Abel et al. found that even after adjusting for age and deprivation differences, women still had higher rates of EPs for colon and rectal cancer compared with men [67]. This may relate to the sex difference in distribution by anatomical site, with symptom profiles likely to vary for CRC subsites.

It has been noted that men are generally less aware of cancer signs and symptoms [46, 68–72]. The 2014 CAM survey found men recalled and recognised fewer signs and symptoms of cancer than women (*p* < 0.001), including recognising persistent change in bowel/bladder habits (*p* < 0.05) [46]. However, although women may have higher levels of awareness it does not mean that they are more likely to have shorter delays from the onset of symptoms until consultation, with the CAM survey showing women reported more barriers to seeing a GP than men (p < 0.001). Delay in women is also evident for other cancers, including breast cancer and gynaecological cancers which have received significantly more publicity and campaign activity than CRC [73–75].

Although men are usually less likely to attend preventative health checks and screening than women, there is generally little evidence that they delay when experiencing actual symptoms of ill-health [76–79]. This also seems to be the case with regard to consultation relating to CRC symptoms and fits with the results presented in this paper. Apart from shorter delays in men seeking medical advice for rectal bleeding [80], no sex differences in consultation have been noted for the most positive predictive symptoms of CRC [81–84]. These include altered bowel habits with a range of other symptoms (including anaemia, weight loss, abdominal pain, diarrhoea and constipation). The more serious the symptoms, such as vomiting, abdominal pain and the presence of obstruction, the shorter the duration of delay in seeking consultation for both men and women, and the greater likelihood that they would be diagnosed through an emergency admission [67, 84, 85].

Getting partner sanction for seeking help is an important factor in early diagnosis [86]; Esteva et al. [85] and Lobchuk et al. [87] both report that women are more likely to push men to see their GP when they have CRC symptoms, whereas women are more likely to wait to see if the symptoms will clear up themselves. Ramos et al. [88] similarly observed that women were more likely not to have discussed their symptoms with their partner prior to their first consultation, which impacts on their early presentation as they procrastinate over whether or not to report the signs.

### Sex differences in stage of cancer at diagnosis

These findings are contrary to those of Nguyen et al. [8], whose systematic review and meta-analysis exploring the implication of sex as a risk factor for advanced neoplasia and colorectal cancer found the pooled relative risk for advanced cancer in men was 1.83 (95% confidence interval, 1.69–1.97). The data from McPhail et al. [26] tends to suggest there is little difference in stage at diagnosis, with more men diagnosed at stage I.

A study by Lyratzopoulos et al. [89], of cancer data covering 2006–2010 from the East of England suggested that although sex was a factor in presenting with more advanced cancer generally, it was not so marked for CRC (including after adjustment for other demographic factors), which is in line with our findings. An analysis of sex disparities in cancer mortality and survival in America also came to the conclusion that though there was a higher death rate in men, this was more closely linked to higher incidence than any other sex, or gender specific factor [35].

### Survival

The Eurocare 4 study found that females had a 2.2% point survival advantage over males, especially in their younger years [90], for cancer deaths due to cancer of the bowel and rectum across Europe. However, our data is more in line with the more recent Eurocare 5 data, which shows a negligible difference between the sexes for colon cancer [91].

### Strengths and limitations

The major strength of this review is that the data has originated from national population based datasets so data are representative of the population.

However, a limitation is that the data were at an aggregate level and results have not been adjusted for other demographic factors that can vary between males and females, with the exception of age where indicated. Therefore, it is uncertain as to what degree some of the sex differences in the data can be accounted for by sex differences in other demographic factors. For instance, variation in the uptake of screening and CRC outcomes have previously been attributed to ethnicity and the individual’s socio-economic circumstances [92–96]; this was not able to be determined from our data. However, previous studies on screening uptake have demonstrated that sex differences exist even after adjustment for other factors [22, 62, 92].

A further limitation to the literature review relates to the current state of play with regard to research on sex and gender differences, with some studies included being of a smaller scale than would be preferable. However, it was felt that for this review the completeness of the overview of the state of play with regard to sex and gender differences warranted their inclusion.

## Conclusion

This is one of the first reviews to start to bring together data on CRC cancer incidence, screening uptake, route to diagnosis, cancer stage and survival specifically from a sex and gender difference perspective. The data show higher incidence rates in males than females. As there are relatively small differences in the data from routes to diagnosis to 5-year survival for males and females, it suggests the higher CRC death rate in males is primarily due to the higher incidence rate. The data show minimal sex differences in survival by stage at diagnosis, which could indicate that there are not significant sex differences in access to and effectiveness of CRC treatment.

Nevertheless, exogenous and/or endogenous sex and gender differences are more apparent for the pathway up to the point of diagnosis of CRC. We highlight that bowel screening uptake is lower in men than women, whilst a higher proportion of CRC cases in women are diagnosed via an EP.

On balance, such differences between men and women mean sex differences in stage distribution are relatively small, but highlight that there could be different approaches focused onto these sex and gendered factors to take to improve early diagnosis across both males and females. Further research could focus on why incidence rates are higher in males and how much the difference is due to modifiable risk factors.

Further understanding of sex differences in CRC could come from analysing data that considers colon and rectal cancers separately, and further breakdowns such as morphology. This may help understand the higher EP rates in females, and how gFOBT/FIT screening is less effective in females than males.

## Abbreviations

ASRs: Age Standardised Rates
BRAF: v-Raf murine sarcoma viral oncogene homolog B
BSS: Bowel Scope Screening
c.f.: Conferatur (Latin) – compare
CAM: Cancer Awareness Measure
CI: Confidence interval
CIMP: CpG island methylator phenotype
CpG: 5’—C—phosphate—G—3
CRC: Colorectal Cancer
EPs: Emergency Presentation
FHb: Faecal haemoglobin
FIT: Faecal immunochemical test
gFOBT: guaiac faecal occult blood test
KRAS: V-Ki-ras2 Kirsten rat sarcoma viral oncogene homolog
PPV: Positive predictive value
TWW: Two week wait
